# Obstetric and perinatal outcomes in pregnancies complicated by diabetes, and control pregnancies, in Kronoberg, Sweden

**DOI:** 10.1186/s12884-019-2269-8

**Published:** 2019-05-07

**Authors:** Anna Stogianni, Lena Lendahls, Mona Landin-Olsson, Maria Thunander

**Affiliations:** 1 Department of Clinical Sciences, Endocrinology and Diabetes, Lund University, Lund, Sweden; 2 Department of Internal Medicine, Endocrinology and Diabetes, Central Hospital, Region Kronoberg, S-351 85 Växjö, Sweden; 3 Department of Research and Development, Region Kronoberg, Växjö, Kronoberg Sweden; 4 Department of Health and Caring Sciences, Linnaeus University, Kalmar, Sweden; 5 Department of Endocrinology, Skåne University Hospital, Lund, Sweden

**Keywords:** Pregestational diabetes, Type 1 diabetes, Type 2 diabetes, Gestational diabetes, Cesarean section, HbA1c, Insulin

## Abstract

**Background:**

Diabetes during pregnancy is an increasingly common metabolic disorder, associated with significantly increased risks for both mother and child. Aim of this study was to compare maternal and perinatal outcomes in women with pregestational (PDM) type 1 (T1DM), type 2 diabetes (T2DM), gestational diabetes mellitus (GDM) and compare these to pregnancies not complicated with diabetes. This study also evaluated a specifically organized care-model mostly involving specialist diabetes nurses.

**Methods:**

Retrospective population-based records review 2009–2012. Rates of maternal (preeclampsia, pre-term delivery, cesarean section (CS)) and fetal outcomes (large for gestational age (LGA), macrosomia, congenital malformations/intrauterine death) were assessed and potential predisposing or contributing factors as maternal age, ethnicity, obesity, weight gain, parity, HbA1c levels, insulin types and doses.

**Results:**

Among 280 pregnancies 48 were PDM, 97 GDM and 135 without diabetes. Within the group with diabetes, early-pregnancy BMI was higher (*p* = 0.0001), pregnancy weight gain lower (11.1 ± 6.7 kg vs 13.1 ± 7.1 kg, *p* = 0.005), more delivered preterm (*p* = 0.0001), by CS (*p* = 0.05), and had more LGA neonates (*p* = 0.06) than the group without diabetes. Among pregnancies with diabetes, GDM mothers gained less weight (9.9 kg vs 13.5 kg) (*p* = 0.006), and rates of CS (*p* = 0.03), preterm deliveries (*p* = 0.001) and LGA (*p* = 0.0001) were not increased compared to PDM; More T1DM infants were LGA, 60% vs. 27% in T2DM. In pregnancies with diabetes obesity, excessive weight gain and multiparity were associated with increased risk of LGA neonates, and mother’s type of diabetes and gestational week were associated with higher rates of CS.

**Conclusion:**

Weight gain during pregnancy was lower in pregnancies with diabetes and prevalence of LGA, CS and preterm deliveries in GDM was not elevated, also for T2DM, except increased prevalence of LGA in T1DM that warrants increased clinical attention, indicating that this model of antenatal diabetes care may have contributed to improved maternal and fetal outcomes.

## Background

Diabetes is a common medical complication of pregnancy, affecting at least 10% of all pregnancies globally [1]. Diabetes during pregnancy can be divided into two subtypes: pregestational diabetes mellitus (PDM) type 1 (T1DM) or type 2 (T2DM), and gestational diabetes mellitus (GDM). GDM is defined as diabetes with first onset during pregnancy [2]. GDM usually constitutes around 90% of all pregnancies complicated by diabetes, while pre-existing diabetes accounts for the remaining 10% [1]. A gradual increase in the prevalence of preexisting diabetes and of GDM has been observed worldwide [1, 3]. Risk factors that may contribute to this development are the advanced age of mothers, the increasing prevalence of obesity, family history of diabetes and modern lifestyle with reduced physical activity, changed dietary habits and smoking [4].

Pregnancies complicated by diabetes are associated with significantly increased risks for both mother and child [5, 6]. Concerning mothers with type 1 diabetes the risks of pre-eclampsia (12.7%), cesarean section (CS) (44.3%) and maternal mortality (0.6%) are significantly higher than in mothers without diabetes [7]. Fetuses of mothers with preexisting diabetes have increased risk for malformations, especially congenital heart diseases and anomalies of the nervous system [8, 9]. This has been shown to be associated with poor metabolic control during the period of organogenesis which occurs in the first trimester of pregnancy and is believed to be due to the negative effects of the hyperglycemic environment on the developing fetus [10]. To reduce adverse maternal and fetal outcomes, it is essential that glucose levels are maintained optimal for being pregnant, which is quite low, and to achieve that very good compliance is required [5].

All this makes it crucial to examine the results of current clinical care and assess if the care provided is sufficient or needs to be changed. We aimed to examine the outcome of maternity diabetes care in the Kronoberg Health Care Region, in southeastern Sweden (185,000 inhabitants) [11]. In Kronoberg diabetes specialist nurses take greater responsibility than is usual in Sweden where most contacts for diabetes in pregnancy occur with specialist physicians. The diabetes nurses in Kronoberg are more involved in the care of both PDM and GDM, than usual, as described below. In other hospitals GDM is often cared for within obstetrical maternal care. For these reasons evaluation of the results and care model is especially important.

### Aims

The primary aim was to evaluate the proportion of perinatal outcomes, as rates of LGA, CS and malformations/intrauterine death, among pregnancies complicated by T1DM or T2DM, and compare the outcome with the GDM group, and with pregnancies not complicated by diabetes.

Secondarily, we aimed for the groups described above to also assess maternal outcomes as the proportion that delivered prematurely, were affected by preeclampsia or neonatal hypoglycemia. All outcomes were examined for potential predisposing or contributing factors as maternal age, parity, marital status, obesity, weight gain, glycated hemoglobin (HbA1c) level during the three trimesters of pregnancy and where applicable, daily doses of long- and short-acting insulin at gestational week 26–28 and at delivery.

## Methods

### Subjects

This is a retrospective population based observational study of all pregnancies complicated by diabetes, PDM, type 1 or type 2, or GDM, who were admitted to the obstetrical ward at Växjö Central Hospital, Region Kronoberg, for antenatal care and delivery from January 1st, 2009 to December 31st, 2012. Data was collected by review of electronic medical records from the Departments of Internal Medicine and Obstetrics and Gynecology (incl. Maternity Health Care) at Växjö Central Hospital and Ljungby Hospital, the only two hospitals in Kronoberg. Data for the GDM population was partly collected during a previously conducted pilot study by midwifery students at Linnaeus University [12]. For comparison, 135 pregnancies without diabetes matched for age, parity, date of delivery were investigated. Växjö Central Hospital is the only Department of Obstetrics in the Kronoberg Region.

The study protocol was approved by the Regional Ethical Review Board at Linköping University, to which Kronoberg is referred for Ethical Reviewing.

### Antenatal care and care model

Pregnant women with PDM were managed according to routine procedures. As soon as pregnancy was confirmed, they were referred to a specialist team with combined experience, where specialist diabetes nurses, midwives, obstetricians and endocrinologists collaborate. The care was delivered within Maternity Health Care/Department of Obstetrics and the Specialist Outpatient Diabetes Clinic of the Department of Internal Medicine at Växjö Central Hospital. The patients from Western Kronoberg start at Ljungby local Hospital and are referred for delivery at the secondary hospital in Växjö. The care model which is publicly evaluated for the first time here was developed over many years in Kronoberg and differs from usual care in the rest of Sweden. The main difference is that pregnant women with diabetes did not routinely have contact with diabetes specialist physicians. Contact was almost exclusively (95%) performed through the diabetes department by specialist diabetes nurses. The obstetrics department did not participate in the diabetes care.

All women with T2DM, who used oral hypoglycemic agents (OHA) before pregnancy were treated with diet alone or shifted to insulin immediately upon contact with the hospital specialist diabetes clinic or later when needed. The use of Metformin during pregnancy was not yet established in the region during the study period. All pregnant women taking insulin were asked to perform blood glucose tests before and after meals and at bedtime daily (7 times) and to adjust the insulin doses accordingly.

The patients were in contact with the diabetes nurses every 2–3 weeks, more frequently during the last trimester of the pregnancy period, and if needed, every week. In Sweden, all women attend prenatal care. Non-attendance is very rare. Glucose goals for the first half of pregnancy were fasting values between 4.5–6.0 mmol/l, post-prandial levels between 6.5–7.5 mmol/l and HbA1c < 52 mmol/mol. For the second half of pregnancy target fasting value was between 4.0–5.5 mmol/l, post-prandial levels < 6.5 mmol/l and HbA1c 36–42 mmol/mol. HbA1c values were measured at 3–4 week intervals [13] with the HemoCue® HbA1c 501 System (Ängelholm, Sweden) [13]. The same glucose goals were applied for PDM and GDM. Glucose values were self monitored blood glucose (SMBG) measured with the patients’own glucometers, which were either FreeStyle Lite®, Abbott One Touch Ultra®, LifeScan or Glucocard X-sensor® OneMed. As for number of extra maternity appointments for women with diabetes this is individual out of need, but in general twice as often, or more, than women without diabetes. Information regarding HbA1c levels and types and doses of insulin was not available for the GDM group.

### Maternal and fetal outcomes

Maternal characteristics assessed included age at conception (years, also dichotomized </≥ 30 years), type and duration of diabetes (T1DM, T2DM, GDM and any vs no diabetes), ethnic/social background (Caucasian, Asian, African), employment was defined as full-time, part-time or other when it included other activities such as being student, on maternity leave or unemployed. Marital status was defined as married/living together vs living alone, previous number of pregnancies. Exercise equivalent to brisk walking 30 min ≥3 times/week were reference to 1-3 times/week or 0 = inactive and miscarriages, previous episodes of GDM, hypertension, smoking (yes/no), type of insulin used (basal, bolus, market substance), insulin quantity required, (mean daily doses at gestational weeks 26–28 and delivery, international units, IE), mean HbA1c (mmol/mol) during the three trimesters of pregnancy, BMI at first visit (early pregnancy BMI), also dichotomized </≥ 25 kg/m^2^, and 30 kg/m^2^; and gestational weight gain (GWG) (kg), dichotomized </≥8 kg. This and the debate and recommendations of recent years of limited weight gain in pregnancies affected by overweight and especially obesity, and three groups of women with diabetes in our study had mean BMI above the range of obesity (< 30 kg/m^2^), are the reasons for using > 8 kg. The recommendation for gestational weight gain for women with these ranges of BMI has been suggested to 5–9 kg, or less if more obese, which has even been described to reduce perinatal complications [14, 15].

The maternal/fetal outcomes investigated were frequency of spontaneous abortions, preeclampsia, perinatal/intrauterine mortality, congenital malformations (all prevalent, defined by ICD-10 codes), pre-term delivery (< 37 weeks), vaginal/cesarean delivery (CS), macrosomia (birth weight > 4500 g), large-for-gestational-age (LGA), birth weight (g), Apgar score at 5 min after birth (< 7/≥7), neonatal hypoglycemia, and prevalence of shoulder dystocia.

Preeclampsia was defined as development of hypertension and proteinuria ≥0.3 g/24 h after 20 weeks’ gestation. LGA was defined as birth weight greater than the 90th percentile for gestational age. Neonatal hypoglycemia was defined as blood glucose level < 2.6 mmol/l during the first 24 h of life. Perinatal mortality was defined as death up to 30 days after childbirth. Multiparity was defined as parity of ≥2 deliveries vs first. LADA (Latent Autoimmune Diabetes in Adults) is by WHO defined as a variant of autoimmune type 1 diabetes, which is islet autoantibody positive, but not insulin requiring at the time of diagnosis [16].

### Statistical analysis

Descriptive data if normally distributed were described by mean and standard deviation, if not normally distributed by median and range (min and max). The t-test and chi 2-test, including Fisher’s exact test, were used for comparison between groups. The non-parametric Mann-Whitney U-test, was used for not normally distributed data. ANOVA was used for comparison of levels of HbA1c during three trimesters. *P*-value ≤0.05 was considered significant. All tests were two-sided. Binary logistic regression (forward enter) was performed with the variables CS, LGA, GWG and congenital malformations/intrauterine mortality (CMD) as dependent variables, yielding Crude Odds Ratios (COR) with 95% CI. All variables were transformed to binary and tried in single regression analysis. Variables that had *p*-values < 0.25 for the specific outcome were included in multiple regression models (stepwise backward Wald), with dependent variables LGA, CS, GWG and CMD for adjusted odds ratios (AOR). Independent variables in each multiple regression can be derived from the tables, or the descriptions in the results section. The statistical analyses were carried out with SPSS (Statistical Package for the Social Sciences, Chicago, Illinois, version 23).

## Results

There were 145 pregnancies complicated by diabetes and 135 pregnancies without diabetes, 280 in total. Of the pregnancies complicated by diabetes, 48 (33%) had PDM and 97 had (67%) GDM. Within the PDM group 37 women (77.1%) had T1DM and 11 (22.9%) had T2DM.

### Maternal outcomes

Baseline data are displayed in Table 1.

**Table 1:** Baseline maternal data, for time of early pregnancy (week 8–12), for pregnant women with either pregestational (PDM) type 1 (T1DM), or type 2 diabetes (T2DM), gestational diabetes mellitus (GDM) or without diabetes in Kronoberg

Maternal data	T1DM	T2DM	P	PDM	GDM	P	Diabetes	No Diabetes	P
N	37	11		48	97		145	135	
Maternal age (years)	29.8 ± 5	33.5 ± 6	0.06	30.7 ± 6	32 ± 5	0.30	31.3 ± 5	31.1 ± 5	0.73
Pregestational BMI (kg/ m^2^)	26.7 (17–44)	34 (18–44)	0.005	28.3 (17–44)	29.7 (16–59)	0.11	29.3 ± 7	25.4 ± 4	0.0001
Multiparous	21 (57%)	8 (73%)	0.49	29 (60%)	66 (67%)	0.46	95 (66%)	81 (60%)	0.39
Previous miscarriages	12 (32%)	4 (36%)	1.0	16 (33%)	*	*	*	43 (32%)	0.76^a^
Previous GDM	2 (5.4%)	7 (64%)	0.0001	9 (19%)	*	*	*	*	*
Duration of diabetes (years)	15 ± 8.1	6 ± 6.3	0.001	13 ± 8.5	*	*	*	*	*
Caucasian	36 (97%)	9 (82%)	0.11	43 (94%)	66 (67%)	0.002	110 (76%)	126 (93%)	0.0001
Smoking	4 (11%)	4 (36%)	0.07	8 (17%)	17 (18%)	1.0	25 (17%)	6 (4.4%)	0.001
Employed	23 (62%)	2 (18%)	0.04	25 (52%)	54 (56%)	0.89	79 (55%)	89 (67%)	0.11
Physical activity ≥1 times/week	28 (76%)	9 (82%)	0.28	31 (65%)	35 (37%)	0.001	66 (46%)	56 (48%)	0.18
Gestational weight gain ≥8 kg	33 (89%)	7 (70%)	0.16	40 (85%)	57 (63%)	0.006	97 (70%)	108 (85%)	0.005
Other endocrine disease (thyroid or adrenal^b^)				9 (21%)				6 (4.4%)	< 0.0001^a^

*T1DM* type1 diabetes mellitus, *T2DM* type 2 diabetes mellitus, *PDM* pregestational diabetes mellitus, *GDM* gestational diabetes mellitus

*Information regarding parity, previous miscarriages, previous GDM and duration of diabetes was not available for the GDM group. Figures are either mean ± SD; median (min-max) or n (%). ^a^Comparison PDM vs no diabetes. ^b^adrenal = primary adrenal insufficiency

### PDM, GDM, T1DM and T2DM

Mean maternal age for women with PDM was 30.7 ± 6 and for the GDM group 32.0 ± 5 years (ns). The T2DM mothers tended to be older, 33.5 ± 6, than those with T1DM, 29.8 ± 5 years. The PDM group gained more weight during pregnancy (*p* = 0.006) in comparison to the GDM group. Within the PDM group, T2DM mothers had higher BMI at admission, 34.0 vs 26.7 kg/m^2^, (*p* = 0.005) compared to T1DM mothers. PDM mothers were more physically active (65%) compared to GDM (37%). More T2DM mothers were unemployed compared to those with T1DM (*p* = 0.04), or GDM, or without diabetes. Median duration of diabetes was higher among the women with T1DM compared to T2DM, 15 ± 8.1 vs 6.0 ± 6.3 years. The incidence of previous GDM was higher in the T2DM (there were only 2 women in the T1DM group with previous GDM, who had initially been assessed as LADA) [16]. More women were of Caucasian background among PDM compared to those with GDM, where 26% had Asian and 7% African background (*p* = 0.002). As expected, a significantly higher number of patients in the PDM group used insulin during pregnancy while for the GDM treatment with diet-only was sufficient for almost 50% to reach the HbA1c goal. There were no maternal deaths, neither among mothers with or without diabetes.

### Any diabetes vs control pregnancies without diabetes

Mothers with any diabetes type, compared to the group without diabetes, had higher early-pregnancy BMI (29.3 ± 7 vs. 25.4 ± 4 kg/m^2^), a larger proportion were smokers (17% vs. 4.4%) and a greater proportion were of Asian origin (19% vs. 0.8%, *p* = 0.0001). The women without diabetes gained more weight during pregnancy (*p* = 0.005).

### Fetal outcomes

#### PDM, GDM, T1DM and T2DM

For maternal and perinatal outcomes see Table 2.

**Table 2:** Complications of pregnancy and perinatal outcomes for women with any type of diabetes, or without diabetes in Kronoberg

Pregnancy outcomes	T1DM	T2DM	P	PDM	GDM	P	Diabetes	No Diabetes	P
N	37	11		48	97		145	135	
Caesarian section (CS)	15 (41%)	5 (46%)	0.16	20 (42%)	24 (25%)	0.03	44 (30%)	25 (19%)	0.05 ^a 0.0001^
Preeclampsia	7 (19%)	3 (27%)	0.68	10 (21%)	*	*	*	10 (7%)	0.15^a^
Birth < 37 weeks gestation	13 (35%)	5 (46%)	0.15	18 (38%)	12 (12%)	0.001	30 (21%)	8 (6%)	0.0001
Large for gestational age (LGA)	22 (60%)	3 (27%)	0.15	25 (52%)	13 (13%)	0.0001	38 (26%)	23 (17%)	0.06
Birth weight > 4500 g	1 (2.7%)	1 (9%)	0.38	2 (4.3%)	6 (6.3%)	1.0	8 (5.6%)	7 (5.2%)	1.0
APGAR score < 7 (at 5th min after delivery)	6 (16%)	2 (18%)	1.0	8 (17%)	4 (4.1%)	0.02	12 (8.3%)	4 (3.0%)	0.07
Neonatal hypoglycemia	12 (32%)	1 (9%)	0.07	13 (27%)	*	*	*	*	*
Congenital malformations	1 (2.7%)	2 (18%)	0.08	3 (6.3%)	*	*	*	4 (2.9%)	0.06^a^
Perinatal/intrauterine mortality	0 (0%)	1 (9%)	0.23	3 (6.3%)	*	*	*	*	*
Shoulder dystocia	1 (2.7%)	0 (0%)	0.16	1 (2.1%)	*	*	*	3 (2.2%)	0.24^a^

T1DM: type1 diabetes mellitus, T2DM: type 2 diabetes mellitus, PDM: pregestational diabetes mellitus, GDM: gestational diabetes mellitus

*Information regarding preeclampsia, neonatal hypoglycemia, congenital malformations, perinatal/intrauterine mortality and shoulder dystocia was not available for the GDM group. Figures are n (%). ^a^Comparison PDM vs no diabetes

The proportion of live born infants born preterm due to either spontaneous onset of labor or termination of pregnancy due to obstetrical reasons was higher in the PDM group, (p = 0,001) and they had a lower APGAR score 5 min after birth (p = 0,02) compared to the GDM. Among children of women with PDM 53% (25/48) were LGA compared to only 13% (13/97) in the GDM group (*p* = 0.0001). There was a higher proportion of caesarian deliveries in the PDM compared to the GDM group (42% vs. 25%) (*p* = 0.03). Of all the mothers 25% (65/262) delivered by CS, 19% (24/127) of those without diabetes. Mother’s type of diabetes (PDM vs GDM, p = 0.03) and gestational week (*p* = 0.01) were associated with CS in women with any type of diabetes, (Table 3). Among mothers with diabetes, only birth week remained associated, AOR (R^2^ 0.08) 3.4 (1.4–7.9) *p* = 0.005; while multiparity, early BMI > 30 kg/m2, ethnicity, birth week, smoking or LGA did not (*p* = 0.18–0.97). Among those without diabetes, for delivery by CS, AOR (R^2^ 0.29) remained significant for early pregnancy BMI > 30 kg/m2 6.2 (1.8–21.5) *p* = 0.004 and multiparity 0.28 (0.10–0.78) *p* = 0.014, but not for ethnicity, birth week, smoking or LGA (*p* = 0.29–1.0) Very few pregnancies, (3/48, 6%) with T2DM and (4/135, 3%) of control pregnancies, were complicated by congenital malformations. There was one cardiac malformation (Ventricle Septum Defect) in the T1DM group (1/37, 2%) and two, one Morbus Down and one retentio testis in the T2DM group (2/11, 18%). Additionally, in the T2DM group, one intrauterine death was observed in week 37 + 3 days. This was the only case of intrauterine death, 1/48, 2% of PDM vs none in the GDM group, and none among the 135 control pregnancies. Early pregnancy BMI > 30 kg/m^2^ was a significant risk factor for congenital malformations and/or intrauterine mortality (*p* = 0.04) and gestational weight gain > 8 kg tended to reach significance as a risk factor (*p* = 0.07). Multiple regression analysis for congenital malformations/intrauterine death among the PDM mothers revealed that only early pregnancy BMI > 30 kg/m2 remained associated with the outcome, AOR 0.85 (0.73–0.99), *p* = 0.03; while GWG, ethnicity, duration of diabetes and mean dose of long acting insulin at gestational week 26–28 lost their significances (*p* = 0.4–1.0).

#### Any diabetes vs control pregnancies without diabetes

Among pregnant women with any diabetes type more delivered preterm (21% vs. 6%, *p* = 0.0001) and by CS (30% vs. 19%, *p* = 0.05) compared to those not complicated by diabetes. There was a tendency towards more LGA neonates among the pregnancies with diabetes (26% vs. 17%, *p* = 0.06).

#### Risk of LGA newborns, Table 4

Frequency of LGA among all mothers, with and without diabetes, was 21.6% (60/278) and among those without diabetes 17.0% (23/135).

**Table 3:** Factors affecting odds for delivery by Cesarean Section (CS) in mothers with any type of diabetes (type 1, type 2 or gestational diabetes) or no diabetes (controls) during pregnancy in Kronoberg

	CS Any diabetes COR(95%CI)	p	CS No diabetes COR(95%CI)	p	CS GDM COR(95%CI)	p	CS T1DM COR(95%CI)	p	CS T2DM COR(95%CI)	p
Diabetes type	2.3 (1.1–4.7)	0.03	–	–	ns	0.47	3.0 (1.4–6.6)	0.006	4.4 (1.2–16.3)	0.03
Maternal age > 30 years	ns	0.45	ns	0.42	ns	0.55	ns	0.39	ns	1.0
Parity (being multipara vs first)	ns	0.51	4.0 (1.6–10.2)	0.003	ns	0.24	ns	0.74	ns	0.50
Marital status (living alone vs not)	ns	0.8	ns	0.80	ns	1.0	ns	1.0	ns	1.0
Ethnicity (Asian or African vs Caucasian)	ns	0.48	ns	0.51	ns	0.77	ns	1.0	ns	1.0
BMI early pregnancy ≥25 kg/m^2^	ns	0.95	ns	0.17	ns	0.59	ns	0.46	ns	1.0
BMI early pregnancy ≥30 kg/m^2^	ns	0.84	3.7 (1.3–10.4)	0.012	ns	0.91	ns	0.43	tendency	0.08
Gestational weight gain ≥8 kg	ns	0.19	ns	0.71	ns	0.35	ns	0.51	ns	0.64
Week of delivery < 37 weeks (vs ≥37 weeks)	3.4 (1.4–7.9)	0.01	ns	0.17	ns	0.98	3.9 (0.94–16)	0.061	tendency	0.08
Smoking (yes vs no)	ns	0.85	ns	0.77	ns	0.88	ns	0.17	ns	1.0
Physically less active (inactive or 1–3 times/week vs > 3 times/week)	ns	0.54	ns	0.96	ns	0.69	ns	0.32	ns	1.0
APGAR 5 min < 7	ns	0.14	ns	0.13	ns	1.0	ns	1.0	ns	1.0
LGA	tendency	0.07	ns	1.0	ns	0.59	ns	0.46	ns	0.50

*COR* crude odds ratio in logistic regression, forward Wald. Gestational diabetes (GDM), type 1 (T1DM) and type 2 (T2DM) diabetes mellitus

**Table 4:** Factors affecting odds for large for gestational age (LGA) in mothers with any diabetes (type 1 (TIDM, type 2 (T2DM) or gestational diabetes (GDM)), or no diabetes, in Kronoberg

	LGA Any Diabetes COR(95%CI)	p	LGA No diabetes COR(95%CI)	p	LGA GDM COR(95%CI)	p	LGA T1DM COR(95%CI)	p	LGA T2DM COR(95%CI)	p
Diabetes type (T1DM vs no diabetes) (T1DM vs GDM)		< 0.0001	–	–	ns	0.47	7.2 (3.3–16.0)^b^ 9.5 (3.9–22.8)	< 0.0001 < 0.0001	ns	0.39
Maternal Age > 30 years	ns	0.40	ns	0.29	ns	0.47	22.1 (3.7–132)	< 0.001	ns	0.78
Parity (being multipara vs first)	2.5 (1.0–5.9)^a^	0.04	ns	0.29	ns	0.46	9.3 (2.0–42)^c^	0.004	ns	1.0
Marital status (living alone vs not)	ns	0.28	4.2 (0.88–20.4)	0.07	8.7 (1.1–69)	0.04	ns	0.78	ns	0.15
Ethnicity (Asian or African vs Caucasian	ns ns	0.16 0.59	ns ns	1.0 1.0	ns ns	0.97 0.25	ns	1.0-	ns ns	1.0
Pregestational BMI ≥25 kg/m^2^	ns	0.39	ns	0.27	ns	0.15	4.0 (0.96–16.5)	0.058	ns	1.0
Pregestational BMI ≥30 kg/m^2^	ns	0.14	ns	0.13	3.2 (0.92–11.4)	0.066	ns	0.14	ns	1.0
Gestational weight gain (GWG) ≥8 kg	3.8 (1.4–10.5)	0.01	ns	0.17	3.8 (0.8–18.4)	0.094	ns	0.17	ns	0.13
Week of delivery < 37 weeks (vs ≥37 weeks)	ns	0.15	ns	1.0	ns	0.72	ns	0.85	ns	0.62
Smoking	ns	0.48	ns	0.85	ns	0.48	ns	0.69	ns	1.0
Physical activity (Inactive or 1-3 times/week vs >3 times/week)	ns	0.14	ns	0.28	ns	0.21	ns	0.58	ns	0.44
APGAR 5 min (< 7 vs ≥7)	ns	0.15	ns	0.67	ns	0.50	ns	0.70	ns	1.0

^a^First pregnancy in mothers with any type of diabetes had lower risk of LGA, COR 0.41 (0.17–0.97), p = 0.04, but it was ns for mothers without diabetes (p = 0.29). ^b^Nagelkerke R^2^ = 0.16. ^c^ Nagelkerke R^2^ = 0.31

Within the PDM group, a higher number of infants in the T1DM group were LGA, 60% (22/37) vs. 27% (3/11) among T2DM, the difference did not reach significance (*p* = 0.15). Only two children, in the PDM group, 4.3% (2/48), were macrosomic at birth. There was a significant association between type of diabetes and risk for LGA newborns, (*p* = 0.0001). Women with T1DM had a 9.5 times higher risk for having LGA children compared to women with GDM (Table 4). In mothers with any diabetes being multipara was associated with a greater risk of having LGA children (*p* = 0.04) as were gestational weight gain ≥8 kg (*p* = 0.01). Women with diabetes treated with insulin were also found to have significantly higher risk for LGA children (*p* = 0.002). In multiple regression analysis, among the mothers with any type of diabetes during pregnancy, the following factors remained significant for LGA after adjustment (R^2^ 0.49) (AOR): diabetes type T1DM 31.3 (7.8–125), *P* < 0.001; multiparity 6.2 (1.8–21), *p* = 0.003: early pregnancy BMI > 30 kg/m2 7.2 (2.0–26), p = 0.003; GWG 3.8 (1.0–14.1), *p* = 0.047 and marital status (living alone) 18.4 (1.7–206), *p* = 0.02; while mother’s age, birth week, smoking and physical activity lost their significance (*p* = 0.10–0.97). In multiple regression analysis the adjusted odds ratio (AOR) for marital status (living alone) tended to be 5.0 (0.92–26), *p* = 0.061, for LGA in the controls, all other factors in the equation (multiparity, early pregnancy BMI > 30 kg, gestational weight gain, birth week, smoking, physical activity lost their significance in the multiple regression.

#### Gestational weight gain

For all mothers, with or without diabetes, GWG was associated with the outcome of LGA, OR 3.2 (1.1–9.5) *p* = 0.05 for ≥8 kg; but not with GWG > 10 kg, *p* = 0.64 or GWG > 12 kg, *P* = 0.47). Early pregnancy BMI > 25 kg/m^2^ and > 30 kg/m^2^ were protective of GWG ≥ 8 kg among mothers with diabetes, OR 0.27–0.43, especially GDM while diabetes type was associated with increased OR of GWG < 8 kg, in T1DM 3.0 (1.4–6.6) *p* = 0.006; and T2DM 4.4 (1.2–16.3) *p* = 0.03, Table 5. In multiple regression analysis only multiparity (vs first) remained significantly associated with GWG (≥8 kg), AOR 0.22 (0.06–0.84), p = 0.03 (R2 0.15) for the controls, LGA, early pregnancy BMI, and ethnicity did not (p 0.09–0.97). Among mothers with diabetes AOR (R^2^ 0.25) for both early pregnancy BMI > 30 kg/m2 0.34 (0.14–0.82) *p* = 0.015; ethnicity (Asian) 0.11 (0.20–0.73) *p* = 0.02; and LGA 3.8 (1.1–12.8) p = 0.03 remained significant, and diabetes type T1DM tended to be 0.30 (0.08–1.1) *p* = 0.06.

**Table 5:** Factors affecting odds for gestational weight gain (GWG) ≥ 8 kg in mothers with any type of diabetes (type 1, type 2 or gestational diabetes), or no diabetes in Kronoberg

	GWG Any diabetes COR(95%CI)	p	GWG No diabetes COR(95%CI)	p	GWG GDM COR(95%CI)	p	GWG T1DM COR(95%CI)	p	GWG T2DM COR(95%CI)	p
Diabetes type		0.020	–	–	0.30 (0.15–0.56)	< 0.0001	4.9 (1.6–15.1)	0.05	ns	0.65
Maternal age > 30 years	ns	0.92	ns	0.57	ns	0.97	ns	0.28	ns	0.88
Parity (being multipara vs first)	ns	0.49	0.25 (0.1–0.90)	0.03	ns	0.32	ns	0.20	ns	1.0
Marital status (living alone vs not)	ns	0.76	ns	0.94	ns	0.98	ns	0.12	ns	0.22
Ethnicity (Asian or African vs Caucasian)	ns ns	0.13 0.29	ns ns	1.0	ns ns	1.0 0.59	ns	1.0	ns	1.0
BMI early pregnancy ≥25 kg/m^2^	0.27 (0.11–0.71)	0.008	ns	0.79	0.31 (0.09–1.0)	0.051	ns	0.86	ns	1.0
BMI early pregnancy ≥30 kg/m^2^	0.43 (0.20–0.90)	0.026	ns	0.29	ns	0.09	ns	0.29	ns	0.88
Week of delivery < 37 weeks (vs ≥37 weeks)	ns	0.46	ns	0.30	ns	0.56	ns	0.65	ns	0.50
Smoking (yes vs no)	ns	1.0	ns	0.31	ns	0.94	ns	1.0	ns	0.78
Physically less active (inactive or 1-3 times/week vs 3 times/week)	ns	0.46	ns	0.86	ns	0.36	ns	0.32	ns	0.5
LGA	3.7 (1.3–10.4)	0.012	ns	0.17	3.8 (0.80–18.4)	0.094	ns	0.17	ns	0.13

*COR* crude odds ratio in logistic regression, forward Wald. Gestational diabetes (GDM), type 1 (T1DM) and type 2 (T2DM) diabetes mellitus

#### Glycemic control and treatment with insulin in PDM, see Table 6

Glycemic control, measured by HbA1c, was lower during the second trimester (*p* = 0.05) and showed a tendency to be lower during the third trimester (*p* = 0.09), in the T2DM pregnancies compared to the T1DM pregnancies, while there was no significant difference during the first trimester (Table 5). In terms of therapy during pregnancy 4/48 (8.3%) patients with PDM made it through pregnancy with diet-only treatment (there were 3 women with T2DM and one woman with T1DM, initially assessed as LADA) and so did 46/97 (47%) in the GDM group. For those with T2DM needing insulin the most common basal insulin used during this period in pregnant women was of NPH type (7/11, 64%), among TIDM the majority used insulin glargine (54.1%) and the remaining either detemir (21.6%) or NPH insulin (8.1%). The most common bolus insulin in the T2DM group was insulin aspart (7/11) whereas most T1DM patients used insulin aspart (57%) or insulin lispro (38%). The main daily dose of basal insulin during pregnancy and at the time for delivery was similar in the two groups. The mean dose of bolus insulin was higher during gestational weeks 26–28 among the T1DM compared to the T2DM mothers. There were no differences found in LGA or CS rates related to the use of, type of or dose of insulin during pregnancy. HbA1c levels or the type or dose of insulin used during pregnancy did not affect the risk for congenital malformations or intrauterine mortality.

**Table 6:** Glycemic control and glucose-lowering therapy for pregnant women with type 1 diabetes (T1DM) or type 2 diabetes (T2DM) in Kronoberg

N	T1DM	T2DM	P
	37	11	
Glycemic control			
HbA1c 1^st^ trimester (mmol/mol)	56 ± 15	49 ± 14	0.14
(Min/max)	32–113	35–73	
HbA1c 2^nd^ trimester (mmol/mol)	49 ± 12	42 ± 6	0.047
(Min-max)	31–82	36–52	
HbA1c 3^rd^ trimester (mmol/mol)	48 ± 11.1	42 ± 7	0.09
(Min-max)	29–80	34–54	
Therapy			
Type of therapy (insulin^a^ vs diet)	36 (97%)	8 (73%)	0.03
Daily dose of basal (weeks 26–28) (units)	28 ± 20.2	25 ± 40.2	0.73
Daily dose of basal at (delivery) (units)	32 ± 21.8	31 ± 39.2	0.92
Daily dose of bolus (weeks 26–28) (units)	48 ± 29.4	26 ± 27.4	0.04
Daily dose of bolus at (delivery) (units)	57 ± 31.3	40 ± 34	0.14

Data are expressed as means ± standard deviation or n (%)

^a^For insulin substances please see the text

## Discussion

In this population based study we examined maternal and fetal outcomes and predisposing factors in all pregnancies complicated by diabetes during 2009–2012 within the whole Health Care Region Kronoberg in southern Sweden. For comparison, a group of pregnancies not complicated by diabetes matched for age, parity and date of delivery, was investigated. We found high mean early pregnancy BMI, as expected in the GDM and T2DM mothers, but also in the T1DM group. GDM has previously been observed to be linked with high maternal BMI [3]. A French study found that the risk was increased in both overweight and obese mothers [17]. In the present study, 75% of the GDM women were overweight with a BMI ≥25 kg/m^2^ and 45% were obese with BMI ≥ 30 kg/m^2^, whereas the control group without diabetes had significantly lower mean BMI in early pregnancy so primary prevention of obesity is important in preventing development of both GDM and obstetric complications [17, 18]. The women with GDM gained less weight during pregnancy, however, compared to those with PDM and to the control group, which is interpreted as an effect of the care given. Among PDM group 54% were overweight and 85% had an excessive gestational weight gain (≥8 kg). The mothers with T2DM tended to be older and had higher BMI, compared to mothers with T1DM (34 vs. 26.7 kg/m^2^). Our findings are in agreement with a review that included papers of both T1DM and T2DM pregnancies 1987–2008 from many countries globally, that also found that women with T2DM were older (33.9 vs. 28.8 years) and heavier (30.2 vs. 24.2 kg/m^2^) compared to women with T1DM [19]. It is noteworthy that even the mean weight of the T1DM mothers in early pregnancy was in the overweight range, in congruence with recent findings that obesity was common in T1DM patients, especially in the women, also from Kronoberg [20].

The incidence of previous GDM, as expected, was significantly higher among T2DM. Women that have had GDM are more likely to develop T2DM than those with normal glucose tolerance during pregnancy, but studies vary in their estimates of risk [21]. A large Australian study of 6253 patients 1971–2003 found a cumulative risk for T2DM of 25.8% 15 years after GDM [21], emphasizing the importance of long-term follow-up of these women and confirms the need for new strategies to engage these women in lifestyle modification programs involving increased physical activity and a healthier diet [22–24].

Our study, in line with other studies worldwide, showed an association between the demographic characteristics of pregnant women and development of GDM. Ethnic origin has been demonstrated to have a large influence on the prevalence of gestational diabetes with mothers of Asian or African origin having respectively, four and two times higher risk to develop GDM, compared to mothers of Caucasian origin [25, 26]. In the current study we found a higher proportion (33%) of non-Caucasians among the pregnant women with GDM in comparison to the PDM (2.8%) and non-diabetic (6.7%) groups. Among non-Caucasian mothers 26% were of Asian and 7% of African descent. This finding is notable considering that there was a total of 9 and 3.4% of Asian and African women respectively who gave birth during 2012 in Sweden, according to the Swedish National Board of Health and Welfare 2012 [27]. A Norwegian study concluded that a possible explanation for these ethnic differences may be that the Asian women are more insulin resistant in early pregnancy compared to Western Europeans, after adjustments for BMI, and show a less sufficient β-cell function [28]. This may indicate that there is a need for more vigilant monitoring of pregnant non-Caucasian women using lower fasting and post-load glucose thresholds to diagnose gestational diabetes [26]. The proportionality we saw between GDM/T2DM and pregestational T1DM are in line with the general proportions of T2DM vs T1DM in Northern Europe [11].

In the present study the mode of delivery was CS in 42% of the women with PDM and 25% of the women with GDM in comparison to only 19% of the women without any diabetes. This was also higher than the average in in Sweden, 16.6% according to the Swedish National Board of Health and Welfare 2012 [27]. As all pregnancies complicated by any type of diabetes, and especially PDM, are followed more frequently with ultrasound, and if the fetal growth is considered abnormally increased a CS is scheduled for gestational week 38. The increased rate of CS was in accordance with the findings of increased rates of CS in all pregnancies complicated by diabetes found in a retrospective study, from Israel [28]. Other factors like excessive gestational weight gain and/or obesity [15, 17], as well as mothers advanced age are also associated with higher odds of CS [29]. Our data showed an association between mother’s gestational week with CS in diabetic pregnancies, but we found no association with any other predisposing factor. The somewhat limited number of pregnancies complicated by PDM in the cohort might be part of the explanation to this (Table 3).

Obesity and excessive weight gain during pregnancy have been associated with increased risk of LGA children [30]. This is in agreement with our study where being obese, having PDM and a weight gain ≥8 kg during pregnancy were all significantly associated with LGA children. The risk for LGA was 9.5 times higher in T1DM pregnancies compared to the GDM pregnancies. Rates of LGA have in previous studies been found to be comparable between T1DM and T2DM [19]**.** In the current study and in accordance with some recent studies [18, 31], we found that LGA rates in pregnancies with T1DM were significantly higher (59%) in comparison to the T2DM (27%). This is also in accordance with increasing prevalence of LGA in T1DM described in a longitudinal Swedish study in which the incidence of LGA was 8 times higher in the T1DM in comparison to the general obstetric population in Sweden over a period of 13 years (1991–2003) [32]. The etiology behind the increased risk of LGA in the T1DM pregnancies is not fully understood but findings point towards the effect of a disturbed maternal lipid metabolism with high levels of Free Fatty Acids (FFAs) and Triglycerides (TG) in the fetal metabolic environment, resulting in LGA neonates independently of maternal BMI and good glycemic control [33], so in T1DM pregnancies, fetal macrosomia is still a significant problem despite modern therapy, why further investigations are warranted [34]**.**

The T2DM group of women had a milder glycemic disturbance compared to the T1DM group, with lower HbA1c at the first visit and throughout pregnancy. This might be attributed to a better β-cell function due to intrinsic characteristics and shorter duration of diabetes [35, 36]. There is no doubt, however, that globally there are many pregnant women with T2DM now, and that research to find ways to better their prognosis is urgent [37]. Despite that level of glycemic control is considered to be the most important risk factor for congenital malformations and perinatal/intrauterine morbidity, and in contrast to other studies [18], we found a higher proportion of congenital malformations in the type 2 group. More specifically, two malformations (one Morbus Down and one retentio testis) and one late intrauterine death were observed in the T2DM group. This must, however, be interpreted with caution due to very few events and the limited number of pregnant women with T2DM in this cohort, but other factors like maternal obesity, might to some extent explain those results. We found that early pregnancy BMI was a significant risk factor. All the women whose pregnancies resulted in these adverse outcomes had a BMI > 35 kg/m^2^ and two of them had a weight gain ≥8 kg during pregnancy (*p* = 0.07) while no other variables reached significance. One might also speculate, however, if the low proportion of adverse outcomes, except for LGA, in our study, is a result of the care providing model with extra involvement of specialized diabetes nurses.

## Conclusions

Obesity in pregnancies complicated by diabetes, especially type 1 diabetes, where obesity is now more prevalent, was found to be associated with increased risk of LGA neonates. That weight gain during pregnancy was lower among the pregnancies complicated by diabetes, and that the frequency of LGA, or other complications, except for delivery by Cesarean Section, was not elevated in the group with gestational diabetes, indicated that this model of antenatal diabetes care delivered mainly by specialist diabetes nurses may have contributed to the improved outcomes for GDM, and for pregestational type 1 and type 2 diabetes, except for level of HbA1c in the last trimester, which could be lower in women with T1DM, as could the prevalence of LGA children. The increased prevalence of LGA in T1DM despite better maternal BMI compared to T2DM, and mostly good glycemic control warrants increased clinical attention and further investigation.

## Abbreviations

CS: Cesarean section
GDM: Gestational diabetes mellitus
LGA: Large for gestational age
PDM: Pregestational diabetes
T1DM: Type 1 diabetes mellitus
T2DM: Type 2 diabetes mellitus
